# Pharmacological Regulation of Neuropathic Pain Driven by Inflammatory Macrophages

**DOI:** 10.3390/ijms18112296

**Published:** 2017-11-01

**Authors:** Norikazu Kiguchi, Daichi Kobayashi, Fumihiro Saika, Shinsuke Matsuzaki, Shiroh Kishioka

**Affiliations:** Department of Pharmacology, Wakayama Medical University, Wakayama 641-0012, Japan; kobay@wakayama-med.ac.jp (D.K.); fumifumi@wakayama-med.ac.jp (F.S.); matsuzak@wakayama-med.ac.jp (S.M.); kishioka@wakayama-med.ac.jp (S.K.)

**Keywords:** cytokine, chemokine, leukocyte, neutrophil, neuroinflammation, allodynia, hyperalgesia, nicotinic acetylcholine receptor

## Abstract

Neuropathic pain can have a major effect on quality of life but current therapies are often inadequate. Growing evidence suggests that neuropathic pain induced by nerve damage is caused by chronic inflammation. Upon nerve injury, damaged cells secrete pro-inflammatory molecules that activate cells in the surrounding tissue and recruit circulating leukocytes to the site of injury. Among these, the most abundant cell type is macrophages, which produce several key molecules involved in pain enhancement, including cytokines and chemokines. Given their central role in the regulation of peripheral sensitization, macrophage-derived cytokines and chemokines could be useful targets for the development of novel therapeutics. Inhibition of key pro-inflammatory cytokines and chemokines prevents neuroinflammation and neuropathic pain; moreover, recent studies have demonstrated the effectiveness of pharmacological inhibition of inflammatory (M1) macrophages. Nicotinic acetylcholine receptor ligands and T helper type 2 cytokines that reduce M1 macrophages are able to relieve neuropathic pain. Future translational studies in non-human primates will be crucial for determining the regulatory mechanisms underlying neuroinflammation-associated neuropathic pain. In turn, this knowledge will assist in the development of novel pharmacotherapies targeting macrophage-driven neuroinflammation for the treatment of intractable neuropathic pain.

## 1. Introduction

Neuropathic pain elicited by damage to or dysfunction of the sensory nervous system severely affects quality of life and is associated with a high economic cost for both the individual and society [1,2,3]. The symptoms of neuropathic pain are sensory hypersensitivity defined by spontaneous pain, hyperalgesia, and allodynia [4,5,6] resistant to standard analgesics [7,8]. Neuropathic pain can be caused by not only physical lesions (e.g., traumatic nerve injury and spinal cord injury) but also other reasons, such as diabetes, chemotherapy, and viral infection [9,10,11], and senior people have a high risk of suffering neuropathic pain [12,13]. It is difficult to treat all types of hypersensitivity with currently available medications, and many patients with neuropathic pain do not receive appropriate treatment [14,15,16]. Thus, there is an urgent need for evidence-based development of novel pharmacotherapies for neuropathic pain.

A number of experimental animal models [17,18,19,20] have contributed to our understanding of the key components of neuropathic pain. In particular, recent studies have suggested that interactions between the nervous and immune systems trigger chronic neuroinflammation resulting in aberrant sensory processing and neuropathic pain [17,21,22]. Upon nerve injury, several cell types, including damaged neurons, demyelinated Schwann cells, and tissue-resident macrophages, produce soluble inflammatory cytokines, chemokines, and damage-associated molecular patterns (DAMPs) that activate surrounding cells [23,24,25,26,27,28] and recruit circulating leukocytes, such as monocytes/macrophages, neutrophils, and lymphocytes, into the site of injury [22,29,30]. Numerous pro-inflammatory cytokines (e.g., interleukin-1β (IL-1β), IL-6, and tumor necrosis factor α (TNFα)) and chemokines (e.g., CC-chemokine ligand 2 (CCL2, also known as monocyte chemoattractant protein-1), CCL3, and CCL4) are released by the infiltrating leukocytes, directly sensitize nociceptors, and alter the processing of nociceptive information by sensory (dorsal root ganglion, DRG) neurons [31,32,33,34]. Several studies using different rodent models have shown that inhibition of these leukocyte functions prevents the pathogenesis of neuropathic pain [29,35,36,37]. Macrophages have especially significant functions in regulating neuroinflammation; consequently, they are considered to be a common peripheral regulator of neuropathic pain [25,30,38].

The contribution of macrophages to neuropathic pain was mainly uncovered in studies of rodents in which macrophages were depleted by toxin treatment or genetic ablation, which may have had other effects on homeostasis [36,37]. Thus, there is a significant knowledge gap between the rodent studies and the human situation that must be filled before these findings can be translated to the clinic. Nonetheless, it may be possible to develop novel therapeutics for neuropathic pain by pharmacological targeting of immunoregulatory systems in animals without genetic abnormalities. In this review, we highlight our current understanding of how macrophages mediate the pathogenesis of neuropathic pain, and how macrophage activity could be modulated to develop new pharmacological approaches for the relief of intractable neuropathic pain.

## 2. Peripheral and Central Sensitization

It is well known that pain sensation is processed by a discriminative set of primary afferent neurons [39,40]. Unmyelinated C-fibers and thinly myelinated Aδ fibers act as nociceptors, while myelinated Aβ fibers are tactile sensors [41,42]. Noxious stimuli such as heat, cold, pressure, and chemicals are converted to electrical activity by distinct cation channels (e.g., transient receptor potential (TRP) channels and sodium channels) that elicit action potentials [43,44,45,46]. Primary afferent neurons producing glutamate or neuropeptides transmit peripheral information to secondary neurons in the spinal dorsal horn [39,40]. During neuropathic pain, the expression and sensitivity of these channels become dysregulated and elicit ectopic activity of nociceptive DRG neurons [46,47,48]. Despite the complexity of the underlying mechanisms, the close relationship between ectopic activity and pro-inflammatory mediators has been noted in several studies [21,22,49,50]. Because many nociceptive DRG neurons express pro-inflammatory cytokine and chemokine receptors that are upregulated after nerve injury, pro-inflammatory molecules can directly sensitize nociceptors, such as TRP channels, in C-fibers leading to hypersensitivity. For example, IL-1β, TNFα, IL-6, CCL2, and CCL3 are well-known enhancers of nociceptor activity [31,32,33,34,51]. Thus, long-lasting neuroinflammation resulting from upregulation of inflammatory molecules by damaged tissue and infiltrating leukocytes can contribute to the ectopic discharge of sensory neurons, resulting in peripheral sensitization.

Prolonged abnormal transmission of pain signaling into the spinal dorsal horn due to peripheral sensitization triggers central sensitization [6,52,53], characterized by increased excitability of pain-processing neurons and activation of glial cells (microglia and astrocytes) [17,54,55,56]. These glial cells have been the focus of increasing attention in the past few decades, and their critical contribution to spinal neuroinflammation underlying neuropathic pain is now well characterized [29,57,58,59,60]. Microglia and astrocytes are activated by several neurotransmitters derived from primary afferent neurons, such as cytokines, chemokines, and nucleotides. Activation of glial cells induces a variety of pro-inflammatory factors that directly or indirectly sensitize pain-processing neurons in the spinal dorsal horn [17,54,55,56,61]. Similar to the peripheral response, typical inflammatory cytokines (IL-1β, TNFα, IL-6), chemokines (CCL2 and CCL3), and growth factors are upregulated in the dorsal horn after nerve injury, and inhibition of these molecules reverses neuropathic pain [62,63,64,65]. These pain-facilitating molecules function to sensitize ionotropic glutamate receptors such as α-amino-3-hydroxy-5-methyl-4-isoxazolepropionic acid (AMPA) receptor and *N*-methyl-d-aspartate (NMDA) receptors [39,55,56,66]. Because AMPA and NMDA receptors play central roles in pain processing in the spinal cord, modulation of their sensitivity by pro-inflammatory mediators derived from activated glial cells is also important for the pathogenesis of neuropathic pain.

## 3. Accumulation of Macrophages in Injured Nerves

Macrophages are a fundamental component of innate immunity and play pivotal roles in the regulation of inflammatory responses and in the host defense against pathogens [67,68]. Upon tissue injury or infection, chemotactic factors, such as chemokines, recruit circulating monocytes to the site of damage. The monocytes then differentiate into macrophages that orchestrate the inflammatory response through cytokine and chemokine production to repair damaged tissues and/or eliminate the source of infection [67,68,69]. In addition, the majority of tissues contain resident macrophages that contribute to the maintenance of tissue homeostasis and the resolution of localized inflammation [70,71,72]. The variety of receptors expressed by tissue-resident macrophages enables their rapid activation by pro-inflammatory cytokines, chemokines, and DAMPs (e.g., high mobility group box 1, HMGB1) produced by damaged cells [71,73,74,75]. Following nerve injury, resident macrophages and Schwann cells synergistically initiate inflammatory responses and elicit long-lasting neuroinflammation through the recruitment of circulating leukocytes to the site of injury [25,29,76].

**Figure 1:**
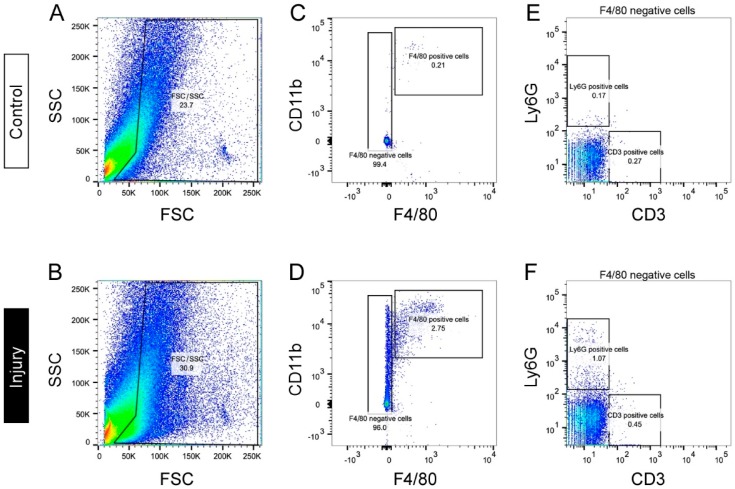
Flow cytometric analysis of leukocytes infiltrating injured nerves. Mice were subjected to partial sciatic nerve ligation, and the sciatic nerves were isolated on day 7 after injury. Representative forward scatter (FSC) versus side scatter (SSC) plots show total events collected from control (**A**) or injured (**B**) sciatic nerves. Representative plots of CD11b versus F4/80 from the gates of each FSC/SSC plot demonstrate that CD11b^+^ F4/80^+^ macrophages are much more abundant in the injured nerves (**D**) than in the control nerves (**C**). Representative plots of Ly6G versus CD3 from the F4/80^−^ population indicate a similar increase in infiltrating Ly6G^+^ neutrophils and CD3^+^ T lymphocytes in the injured (**F**) compared with the control (**E**) nerves.

Neutrophils and lymphocytes also infiltrate into the sites of peripheral nerve injury in rodents [37,77,78,79,80], and depletion of either cell types using experimental drugs or genetic ablation significantly reduces neuropathic pain resulting from neuroinflammation [35,36,37,81,82]. Figure 1 shows a typical analysis of infiltrating macrophages, neutrophils, and T lymphocytes in the injured and contralateral sciatic nerves of mice 1 week after partial sciatic nerve ligation by flow cytometry using well-characterized marker of each leukocyte. An increased number of CD11b^+^ cells was seen in the injured nerve compared with the intact nerve. This population contains F4/80^+^ macrophages, while the F4/80^−^ population included Ly6G^+^ neutrophils and CD3^+^ T lymphocytes. The abundance of each cell type is clearly higher in the injured than in the intact nerve. Importantly, macrophages make up the largest population of infiltrating cells, supporting the notion that they may play a central role in the regulation of neuroinflammation.

Macrophage accumulation is consistently observed in rodent models of experimental neuropathic pain [38], including that resulting from physical nerve damage (e.g., partial sciatic nerve ligation and chronic constriction injury) [83,84,85], diabetes, and chemotherapy (paclitaxel and vincristine) [86,87,88,89]. Notably, targeted inhibition or depletion of macrophages prevents neuroinflammation and pain hypersensitivity in these neuropathic pain models [36,88,90,91]. Collectively, these studies strongly support a critical role for macrophages in peripheral neuroinflammation leading to neuropathic pain.

## 4. Macrophage Polarization and Neuroinflammation

Macrophages are remarkably plastic and can be polarized towards some functionally distinct phenotypes [67,68,92]. Molecules known as “pathogen-associated molecular patterns,” such as bacterial lipopolysaccharides (LPS), and interferon-γ, a cytokine produced by T helper type 1 (Th1) cells, induce the differentiation of classically activated (M1) macrophages that facilitate and exacerbate inflammation [93,94]. In contrast, Th2 cytokines (IL-4 and IL-13) promote differentiation into alternatively activated (M2) macrophages, which suppress inflammation and promote its resolution [75,95]. M1 macrophages are characterized by high expression of pro-inflammatory cytokines such as IL-1β, IL-6, TNFα, and receptors such as Toll-like receptor 4 (TLR4, the LPS receptor), major histocompatibility class II (MHC II), and CD86 [75,93,94]. M1 macrophage-dominant molecules are preferentially upregulated through signaling via signal transducer and activator of transcription 1 (STAT1) and interferon regulatory factor 5 (IRF5) pathways [75,94,96,97]. In contrast, M2 macrophages abundantly express the anti-inflammatory cytokine IL-10 and the scavenger receptors CD163 and CD206 (mannose receptor C type 1) [75,94]. Unlike M1 macrophages, the dominant signaling pathways are via STAT6 and IRF4 in M2 macrophages [97,98]. Chemokine expression patterns also differ between M1 and M2 macrophages [92,93,99,100]; thus, CCL2, CCL3, CCL4 and CCL5 are highly produced by M1 macrophages, whereas CCL18, CCL22 and CCL24 are abundantly expressed by M2 macrophages [99,100,101,102].

The soluble mediators produced by M1 and M2 macrophages are functionally antagonistic [94,97], and the balance between M1 and M2 macrophages is therefore closely related to the progression of inflammatory disorders, including neuropathic pain [103,104]. Prolonged activation of M1 macrophages has been reported to drive non-resolving neuroinflammation that underlies the pathogenesis of neuropathic pain [103,104,105]. Under normal conditions, tissue-resident macrophages are predominantly M2-like and function to protect the peripheral nervous system from damage and to maintain tissue homeostasis [70,72]. However, when the balance tilts in favor of M1 macrophages at the population level, the upregulated expression of M1-dominant molecules has diverse effects on the surrounding cells [75,92,93]. In addition, inflammatory chemokine secretion recruits circulating leukocytes into the inflamed tissue, resulting in long-lasting inflammation [67,69,106]. Since macrophage polarization and activation can be diverse in response to environmental cues [107,108], inhibition of M1 macrophage differentiation has been proposed as a pharmacotherapy for the treatment of neuroinflammatory diseases such as neuropathic pain.

## 5. Roles of Cytokines and Chemokines in Neuropathic Pain

Cytokines and chemokines regulate many aspects of the immune response, including cell-to-cell interactions [109,110,111,112]. IL-1β, IL-6 and TNFα are well-characterized M1 macrophage-dominant cytokines and enhancers of inflammation [92,99,101]. Emerging evidence suggests that these cytokines are upregulated in injured nerves and may contribute significantly to neuropathic pain [17,21,29,113,114]. These cytokines are also produced by Schwann cells and infiltrating leukocytes, suggesting indicating that interactions between these cell types plays a role in injury-induced neuroinflammation [22,25,30,49]. Indeed, upregulation of pro-inflammatory cytokines has been observed in diverse neuropathic pain models (e.g., nerve ligation, diabetes, and chemotherapeutics) [17,38,56,115], and inhibition of their functions using neutralizing antibodies, receptor antagonists, intracellular signaling pathway inhibitors, or genetic ablation significantly reduce hypersensitivity associated with neuroinflammation [23,29,89,116,117,118].

Chemokines are small chemotactic cytokines that also play fundamental roles in neuroinflammation [110,119,120]. These molecules recruit circulating leukocytes into the inflamed tissue and activate various cell types via engagement of cell surface receptors [69,120]. Of the four chemokine families, CC-chemokines are the most well-established regulators of neuropathic pain [30,121,122,123]. In particular, many studies have demonstrated that CCL2 is upregulated in macrophages and damaged Schwann cells following nerve injury [26,51,124,125], and pharmacological or genetic inhibition of CCL2 and/or its receptor CCR2 markedly alleviates neuropathic pain [126,127,128].

**Figure 2:**
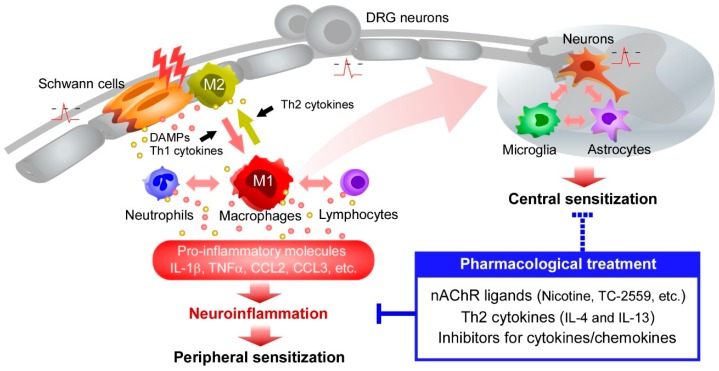
Generation of neuropathic pain by macrophage-driven inflammation in the peripheral nervous system. After nerve injury, activated resident cells (Schwann cells and macrophages) produce soluble factors such as damage-associated molecular patterns (DAMPs) that activate nearby cells and recruit circulating leukocytes (macrophages, neutrophils, and lymphocytes) to the site of injury. Macrophages are the most abundant infiltrating leukocyte population and are thought to play a central role in regulating peripheral neuroinflammation. Tissue-resident and infiltrating leukocytes communicate through the release of pro-inflammatory mediators such as cytokines and chemokines, which convey nociceptive information to dorsal root ganglia (DRG) neurons. Persistent ectopic activity of DRG neurons induces central sensitization characterized by the enhanced activity of pain-processing neurons and the activation of microglia and astrocytes. Pharmacological targeting of macrophages or macrophage-derived pro-inflammatory molecules by nicotinic acetylcholine receptor (nAChR) ligands, Th2 cytokines, and inhibitors of cytokines and chemokines can suppress macrophage-driven neuroinflammation after nerve injury. The reduction in neuroinflammation improves both peripheral and central sensitization and alleviates intractable neuropathic pain.

Recently, CCL3 (macrophage inflammatory protein-1α, MIP-1α) and CCL4 (MIP-1β) have been shown to be upregulated in macrophages and Schwann cells following nerve injury, and to play critical roles in orchestrating long-lasting neuroinflammation in the peripheral nervous system [23,30,123,129]. Because their receptors (CCR1 and CCR5) are also expressed on macrophages and Schwann cells, CCL3 and CCL4 can act in an autocrine fashion to drive intracellular signaling for upregulation of inflammatory molecules such as IL-1β. Accordingly, blockade of CCL3, CCL4, CCR1, or CCR5 function with neutralizing antibodies or inhibitors prevents the upregulation of IL-1β and other M1-dominant cytokines and thus reduces neuropathic pain [23,129]. CCL5 also contributes to the upregulation of M1-dominant cytokines and the recruitment of circulating monocytes, neutrophils, and T lymphocytes to the site of injury [130]. Previous reports have shown that these chemokine receptors are expressed on DRG neurons [33,34,51,131], indicating that CC-chemokines can not only activate inflammatory cells but also directly sensitize nociceptive neurons and enhance pain processing. The CXC-chemokine ligand 2 (CXCL2, also known as macrophage inflammatory protein-2, MIP-2) plays a pivotal role in generating neuropathic pain by mediating interactions between macrophages and neutrophils [35]. CXCL2 and its receptor CXCR2 are upregulated in both cell types in the environment of injured nerves [35]. Neutralizing anti-CXCL2 antibodies or CXCR2 antagonists abolish neutrophil and macrophage infiltration and the upregulation of cytokines underlying neuropathic pain [35]. Therefore, inflammatory cytokines and chemokines derived from M1 macrophages and surrounding inflammatory cells could be novel therapeutic targets for neuropathic pain associated with neuroinflammation (Figure 2). Given that pharmacological inhibitors (e.g., neutralizing antibodies and small compounds) of these key mediators are currently used for inflammatory diseases in the clinic [132,133,134,135], further investigations can facilitate developing effective medications for neuropathic pain.

## 6. Phenotypic Shift of Macrophages by Cytokines

Since M1 macrophages produce many of the key cytokines and chemokines underlying neuropathic pain [30,99,103,122], modulation of macrophage polarization is likely to have a major effect on the pathogenesis of neuropathic pain. Several lines of evidence indicate that IL-4 and IL-13 polarize macrophages toward an M2 phenotype by binding to their receptors, which are composed of heteromeric complexes IL-4 receptor α (IL-4Rα)/common γc chain and IL-4Rα/IL-13Rα1, respectively [136,137]. Downstream signaling through these receptor complexes is mediated by phosphorylation of STAT6 and activation of IRF4, which upregulates the expression of M2-dominant molecules [97,98]. As mentioned, the progression of inflammatory diseases is strongly influenced by the balance between M1 and M2 macrophages [94,138,139]; accordingly, treatment with Th2 cytokines can improve the severity of many diseases by inducing a phenotype shift from the M1 toward the M2 phenotype [140,141,142]. These findings suggest that pharmacological targeting of macrophage polarization using Th2 cytokines may also improve neuropathic pain.

Expression level of IL-4Rα is increased upon nerve injury, mainly due to elevated production by infiltrating macrophages [103]. Notably, addition of IL-4 to LPS-stimulated macrophages in vitro or to isolated injured nerves ex vivo decreases the production of M1-dominant molecules (IL-1β and CCL3) and increases that of M2-dominant molecules (IL-10 and CD206) through activation of STAT6 [103]. Consistent with this, administration of IL-4 to injured nerves may directly shift the phenotype of induced macrophages from M1 toward M2, resulting in the relief of neuropathic pain [103]. Moreover, IL-13 shows similar effects to IL-14, and it also suppresses neuropathic pain by shifting the balance towards M2 macrophages [104]. These recent findings substantiate previous reports demonstrating that neuropathic pain is abolished or attenuated by inhibition of M1-dominant molecules or enhancement of anti-inflammatory cytokines such as IL-10 [17,22,23,30,130,143,144]. It is important to note that IL-4 and IL-13 are beneficial not only during the early phase of nerve injury but also during the middle/late phases, which is similar to the effects of ligands of α4β2 nicotinic acetylcholine receptor (nAChR) [103,104,145]. Since these effects are independent of the pathophysiological stage, infusion of Th2 cytokines may be an attractive pharmacotherapy of neuroinflammation-induced neuropathic pain, particularly since exogenous Th2 cytokines are unlikely to cause severe adverse effects (Figure 2).

## 7. Inhibition of Macrophages by Nicotinic Acetylcholine Receptors

Although therapeutic agents that act on pro-inflammatory mediators have had considerable success in the clinic [146,147,148,149], the effectiveness of single molecularly targeted inhibitors is sometimes limited [150,151,152,153], implying that combination multi-targeted therapy may be necessary for successful treatment of inflammatory diseases.

The peripheral immune system is modulated by the central nervous system [146,154]. Emerging evidence indicates that vagus nerve activation can suppress inflammation through nAChR-mediated inhibition of macrophage function [155,156,157,158]. Consistent with this, nicotine treatment improves a variety of intractable inflammatory diseases in rodents [146,156,157,159,160]. nAChRs are ligand-gated cation channels consisting of homo- or hetero-pentameric complexes formed from distinct subunits [161,162]. The first subtype to be demonstrated to have inhibitory effects on macrophages was α7 [155]. Nicotine treatment can inhibit the production of inflammatory cytokines by LPS-stimulated macrophages via activation of α7 nAChR signaling. Moreover, a number of studies have reported that nicotine treatment suppresses various inflammatory diseases by inhibiting production of M1 macrophage-dominant factors such as IL-1β, TNFα, and HMGB1 [156,159,160]. In contrast, neither nicotine nor other α7 nAChR ligands affect the production of M2 macrophage-dominant molecules such as IL-10 [146,156]. Although the majority of reports focused on the anti-inflammatory property of α7 nAChR, some studies have found that the α4β2 nAChR subtype has similar effects, depending on the tissue injury or disease [163,164].

Macrophages and neutrophils that accumulate around injured nerves express both α7 and α4β2 nAChRs [145,165], and administration of nicotine to the site of injury suppresses upregulation of M1-dominant cytokines and neuropathic pain [165]. Importantly, the suppressive effects of nicotine are reversed by selective antagonism of the α4β2, but not α7, subtype, revealing the dominant role of α4β2 nAChR in the regulation of inflammatory macrophage-driven neuropathic pain [145]. Furthermore, engagement of α4β2 nAChR by either local or systemic administration of the selective ligand TC-2559 markedly reduced neuropathic pain [145]. TC-2559 treatment of LPS-stimulated macrophages suppressed the expression of IL-1β and CCL3 by M1 macrophages by inhibiting the phosphorylation of STAT3 [166], a key transcription factor for these cytokines [167,168]. The observation that α4β2 nAChR ligands are effective in reducing neuropathic pain in rodents during the early, middle, and late phases of nerve injury suggests that α4β2 nAChR expressed on macrophages could be a novel pharmacological target (Figure 2).

## 8. Conclusions

In this review, we have highlighted the critical roles of macrophages in regulating neuropathic pain caused by peripheral neuroinflammation. A growing body of evidence supports the contribution of infiltrating leukocytes, including macrophages, to the pathogenesis of neuropathic pain, as demonstrated in several animal models. Since macrophages play a central role in regulating peripheral sensitization, cytokines and chemokines derived from these cells are potential targets for novel therapeutics, particularly as combination therapies. Approaches that use physiological modulators of macrophage function (e.g., nAChR ligands and Th2 cytokines) may be more effective and have greater safety margins because they function as endogenous immunoregulators. Most importantly, these approaches are effective at the early, middle, and late phases of neuropathic pain after nerve injury.

Although several molecules have been shown to prevent or dramatically relieve neuropathic pain in rodent models, their efficacy has not yet been evaluated in humans. Most rodent studies have employed single experimental models, and there are major systemic and anatomical differences between rodents and primates. The evidence that macrophages contribute to the pathogenesis of neuropathic pain in humans is obviously more circumstantial because of the difficulty in performing functional studies in humans [169,170,171,172]. Among the key questions that remain are whether infiltrating leukocytes mediate the sensory abnormality, and whether neuroinflammation parallels neuropathic pain in patients. The basic components of the inflammatory response are similar in rodents and primates; however, further studies will be necessary to identify the mechanisms that regulate neuroinflammation-associated neuropathic pain in humans. Development of novel evidence-based pharmacotherapies that target macrophage-driven neuroinflammation will undoubtedly open up a new avenue for the treatment of intractable neuropathic pain.
